# Supplementary material for: EnvC Homolog Encoded by Xanthomonas citri subsp. citri Is Necessary for Cell Division and Virulence
Source: Microorganisms. 2024 Mar 29;12(4):691. doi: 10.3390/microorganisms12040691 (PMC11051873; doi:10.3390/microorganisms12040691)

Figure S1. Deletion of the central portion of gene XAC0024 from *X. citri* by double-joint PCR. (A) Schematic representation of the 593 bp deletion in the central portion (fragments A-B and C-D has an overlap of 18 nucleotides); (B) Electrophoresis in 1.0% agarose gel. M: GeneRuler™ 1kb DNA Ladder (Fermentas).

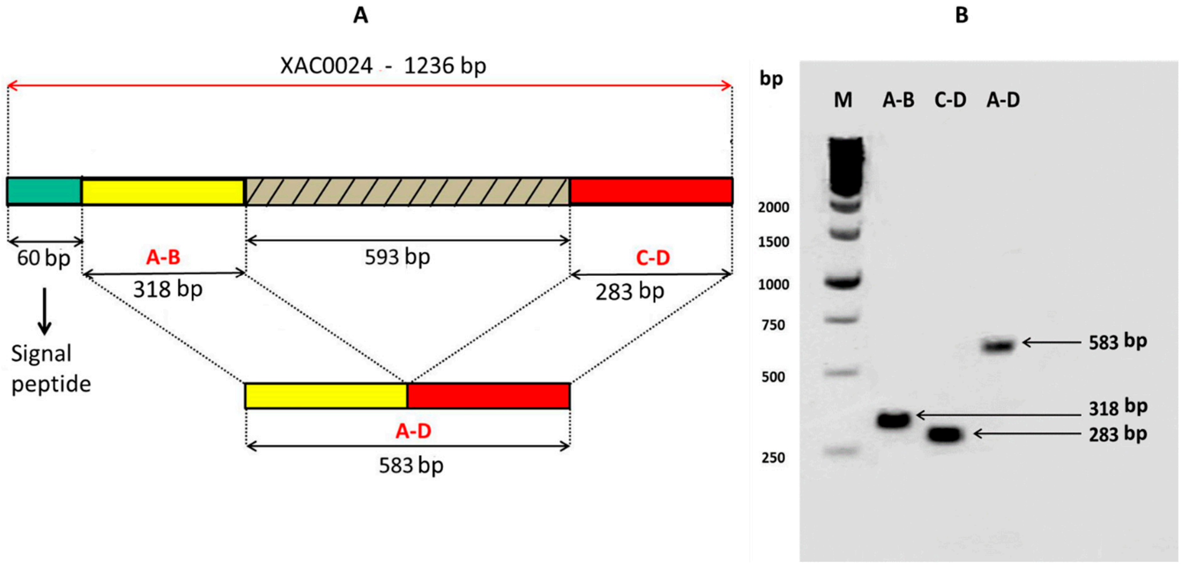

Supplement: Supplementary file 1 [file microorganisms-12-00691-s001.zip › Supplementary Figure S1.pdf]
