# Supplementary material for: EnvC Homolog Encoded by Xanthomonas citri subsp. citri Is Necessary for Cell Division and Virulence
Source: Microorganisms. 2024 Mar 29;12(4):691. doi: 10.3390/microorganisms12040691 (PMC11051873; doi:10.3390/microorganisms12040691)
Supplement: Supplementary file 1 [file microorganisms-12-00691-s001.zip › Supplementary Figure S2.pdf]

Figure S2: Nucleotide sequence alignment between XAC0024 from *X. citri* and its homologue XCC0022 from *X. campestris*. The alignment was performed using the NCBI - Blastn (https://blast.ncbi.nlm.nih.gov).

```
Query: NC_003919.1:c27117-25882 Xanthomonas citri pv. citri str. 306, complete sequence Query ID: lcl|Query_53921 Length: 1236

>NC_003902.1:c26998-25781 Xanthomonas campestris pv. campestris str. ATCC 33913 chromosome, complete genome
Sequence ID: Query_53923 Length: 1218
Range 1: 1 to 1218

Score:1368 bits(1516), Expect:0.0,
Identities:1034/1218(85%),  Gaps:0/1218(0%), Strand: Plus/Plus

Query  19    GTGCTGGCCTGCACCTTGCTGGGCAGCATGGGCGCAAGCGCGCAGAGCCAGCGCGAGACC  78
Sbjct  1     GTGTTCGCCTGCACGCTGCTGGCCGGCACCAGCGCCGGGGCGCAGAGCCAGCGCGAGGCC  60

Query  79    GAGCGCAAATTGCAGCAGCTGCGCGATGAGCTCAAGACCATCAGCGCCGATCGACGCGAA  138
Sbjct  61    GAGCGCAAGCTGCAGCAATTGCGCGATGAACTGAAAACCATCAGCGCCGACCGCCGCGAC  120

Query  139   CTGGAAGGCAAGCGCGGCACCGCCGCGCAACAGTTGCGCCAGGCCGACGAGAAAGTGGCC  198
Sbjct  121   CTGGAAGGCAAGCGCGGCACCGCCGCCCAGCAGCTGCGCCAGGCCGATGAAAAAGTCGCC  180

Query  199   AAGACCGCACGTGCATTGAGCGAGACCGAAGCGGCGATGCGCGCGCAGGAACAGCATCTG  258
Sbjct  181   AAGACCGCACGTGCGCTGAGCGAGACCGAAACCGCGCTGCGCACGCACGAACAGAAACTC  240

Query  259   TCCACCCTGCAGCAGGAACGCGCGCAATTGCAGCGCGGCCTGCAGAACCAGCGCGCGCAG  318
Sbjct  241   TCCGAACTGCAGCAGCAGCGCGCGCAGCTGCAGCGTGGCCTGCGCGAGCAACGCGTGCAG  300

Query  319   CTGGCGGCGTTATTGCGCGCCGCCGATCAGGTGGGCCGCAATGCACCGCTGAAGGTGCTG  378
Sbjct  301   TTGGCCGCACTGCTGCGCGCCGCCGACCATGTGGGCCGCAACGCGCCGCTGAAAGTGCTG  360

Query  379   CTGTCGCAGGACACGGTGGGCGATGCCACGCGTATGCTGGCCGATCACCGCTATGTGCAA  438
Sbjct  361   TTGTCACAAGACACCGTGGGCAATGCCACGCGCATGCTGGCCGACCACCGCTACGTGCAG  420

Query  439   AACGCACGCGCGCAGCGCATCCACGCCTTGACCACGCAACTGGATGCCCTGGCGACAGTG  498
Sbjct  421   AGCGCACGCGCGCAGCGCATCCAGGGGCTCACCACGCAACTGGAAGCACTGACCCAGGTC  480

Query  499   GAACAGGACATCGCCACCCGGCGCCAGGCGCTGGATGCCGCGCGTGCGCAGCAAAAAGCG  558
Sbjct  481   GAGCAGCAAATCACCGAGCGGCGCCAGGCGCTGGACGCCGCACGCGCACAACAAAAAGCA  540

Query  559   CAGGCGGCCACGTTGCAGAAGGATCGCTCGCAACAGGCGGCCACCGTCGCGCAGCTGGAC  618
Sbjct  541   CAGGCCTCGTCGCTGCTGAAAGACCGCTCGCAGCAGGCCGCCACCGTTGCGCAACTGGAC  600

Query  619   GATCGCTACAAACAGCGCGCCGAGCGCGAAAAGGCAATCGGCCAGGACGCCAAGGCGCTG  678
Sbjct  601   ACCCGCTATCAACAGCGCGCCGAGCGCGAGAAAGCCCTGGGCCAGGATGCCAAGGCGCTG  660

Query  679   GAACAACTGCTCGCCAATCTgcgcgcggccgccgccaaggccgaagccgaacggcgcgct  738
Sbjct  661   GAGCAGTTGCTGGCCAATCTGCGTGCAGCCGCGGCCAAGGCAGAAGCCGAGCGCCGCGCG  720

Query  739   gccgccagacgcgccgccgccgaagccgccgcACAGGCCAAGCGCAGCAAAACCGAGCGT  798
Sbjct  721   GCGGCCAAGCGTGCGGCCGCCGAAGCCGCCGCGCAGGCCAAGCGCGGCAAAACCGATCGC  780

Query  799   TCCGATCGCCCCGGCAAGACGCCATCCAAGGTAGTCGCCAACGCACCGGCCCCCAAGGTC  858
Sbjct  781   CCGGAACGCCCCGGCAAGACACCGCCCAAGGTCGTGGCCAACGCACCGGCGCCAAAGGTC  840

Query  859   GGTGGGCTCAGCTGGCCGGTGGCGGGCAATCTGCTGGCGCGCTTCAATGCCACCTTGCCC  918
Sbjct  841   GGTGGCCTGAGTTGGCCGGTGTCGGGCAACCTGCTGGCGCGCTTCAACGCCACGCTGCCG  900

Query  919   GATGGCCACACCAGCAAGGGCGTGCTGATCGGCGCGCCCAAGGGCACCACCGTGACTGCA  978
Sbjct  901   GATGGCCATACCAGCAAGGGCGTGCTGATCGGCGCGCCCAAGGGCAGCACGGTCACCGCG  960

Query  979   GTGGCCGATGGCACGGTGGTGTTTTCCGACTGGATGACCGGCTACGGCATGATCCTGATC  1038
Sbjct  961   GTGGCCGATGGCACGGTGGTGTTTTCCGACTGGATGACCGGCTACGGCATGATCCTGATC  1020

Query  1039  GTGGACCACGGCAACGGCTACATGAGCCTGTACGCGCATAACGACACCTTGCTGCGCGAT  1098
Sbjct  1021  GTGGATCACGGCAACGGCTACATGAGCCTGTACGCGCACAACGACACCTTGCTGCGCGAT  1080

Query  1099  GCCGGCGCAACGATCAAACGTGGCGACGCGGTGGCCAAGGTCGGCAGTTCGGGCGGGCAG  1158
Sbjct  1081  GCGGGCGCCAGCATCAAGCGTGGCGAGGCCGTGGCCAAGGTCGGCAGTTCGGGCGGGCAG  1140

Query  1159  GGCGTGCCGGCGCTGTACTTCGAATTGCGTCGCAACGGGCAGCCGGTGGATCCATCGAGC  1218
Sbjct  1141  GGTGTGCCGGCGCTGTACTTCGAGCTGCGTCGCAACGGCCAGCCAGTGGATCCTTCGAGC  1200

Query  1219  TGGCTGCAACGCCGCTGA  1236
Sbjct  1201  TGGCTGCAGCGCCGCTGA  1218
```
