# Supplementary material for: EnvC Homolog Encoded by Xanthomonas citri subsp. citri Is Necessary for Cell Division and Virulence
Source: Microorganisms. 2024 Mar 29;12(4):691. doi: 10.3390/microorganisms12040691 (PMC11051873; doi:10.3390/microorganisms12040691)
Supplement: Supplementary file 1 [file microorganisms-12-00691-s001.zip › Supplementary Figure S3.pdf]

Figure S3: Protein sequence alignment between XAC0024 from *X. citri* and its homologue XCC0022 from *X. campestris*. The alignment was performed using the NCBI - Blastp (https://blast.ncbi.nlm.nih.gov).

```
Query: CEE16095.1:22-432 conserved exported hypothetical protein [Xanthomonas citri pv. citri] Query ID: lcl|Query_30397 Length: 411

>NP_635417.1 hypothetical protein XCC0022 [Xanthomonas campestris pv. campestris str. ATCC 33913]
Sequence ID: Query_30399 Length: 405
Range 1: 1 to 405

Score:610 bits(1574), Expect:0.0,
Method:Compositional matrix adjust.,
Identities:359/405(89%), Positives:378/405(93%), Gaps:0/405(0%)

Query  7    VLACTLLGSMGASAQSQRETERKLQQLRDELKTISADRRELEGKRGTAAQQLRQADEKVA  66
            + ACTLL    A AQSQRE ERKLQQLRDELKTISADRR+LEGKRGTAAQQLRQADEKVA
Sbjct  1    MFACTLLAGTSAGAQSQREAERKLQQLRDELKTISADRRDLEGKRGTAAQQLRQADEKVA  60

Query  67   KTARALSETEAAMRAQEQHLSTLQQERAQLQRGLQNQRAQLAALLRAADQVGRNAPLKVL  126
            KTARALSETE A+R  EQ LS LQQ+RAQLQRGL+ QR QLAALLRAAD VGRNAPLKVL
Sbjct  61   KTARALSETETALRTHEQKLSELQQQRAQLQRGLREQRVQLAALLRAADHVGRNAPLKVL  120

Query  127  LSQDTVGDATRMLADHRYVQNARAQRIHALTTQLDALATVEQDIATRRQALDAARAQQKA  186
            LSQDTVG+ATRMLADHRYVQ+ARAQRI  LTTQL+AL  VEQ I  RRQALDAARAQQKA
Sbjct  121  LSQDTVGNATRMLADHRYVQSARAQRIQGLTTQLEALTQVEQQITERRQALDAARAQQKA  180

Query  187  QAATLQKDRSQQAATVAQLDDRYKQRAEREKAIGQDAKALEQLLANLRAAAAKAEAERRA  246
            QA++L KDRSQQAATVAQLD RY+QRAEREKA+GQDAKALEQLLANLRAAAAKAEAERRA
Sbjct  181  QASSLLKDRSQQAATVAQLDTRYQQRAEREKALGQDAKALEQLLANLRAAAAKAEAERRA  240

Query  247  AARRAAAEAAAQAKRSKTERSDRPGKTPSKVVANAPAPKVGGLSWPVAGNLLARFNATLP  306
            AA+RAAAEAAAQAKR KT+R +RPGKTP KVVANAPAPKVGGLSWPV+GNLLARFNATLP
Sbjct  241  AAKRAAAEAAAQAKRGKTDRPERPGKTPPKVVANAPAPKVGGLSWPVSGNLLARFNATLP  300

Query  307  DGHTSKGVLIGAPKGTTVTAVADGTVVFSDWMTGYGMILIVDHGNGYMSLYAHNDTLLRD  366
            DGHTSKGVLIGAPKG+TVTAVADGTVVFSDWMTGYGMILIVDHGNGYMSLYAHNDTLLRD
Sbjct  301  DGHTSKGVLIGAPKGSTVTAVADGTVVFSDWMTGYGMILIVDHGNGYMSLYAHNDTLLRD  360

Query  367  AGATIKRGDAVAKVGSSGGQGVPALYFELRRNGQPVDPSSWLQRR  411
            AGA+IKRG+AVAKVGSSGGQGVPALYFELRRNGQPVDPSSWLQRR
Sbjct  361  AGASIKRGEAVAKVGSSGGQGVPALYFELRRNGQPVDPSSWLQRR  405
```
