# Supplementary material for: EnvC Homolog Encoded by Xanthomonas citri subsp. citri Is Necessary for Cell Division and Virulence
Source: Microorganisms. 2024 Mar 29;12(4):691. doi: 10.3390/microorganisms12040691 (PMC11051873; doi:10.3390/microorganisms12040691)
Supplement: Supplementary file 1 [file microorganisms-12-00691-s001.zip › Supplementary Figure S4.pdf]

Figure S4: Protein sequence alignment between XAC0024 from *X. citri* and EnvC from E. *coli*. The alignment was performed using the NCBI - BLASTP (https://blast.ncbi.nlm.nih.gov).

```
Query: CEE16095.1:22-432 conserved exported hypothetical protein [Xanthomonas citri pv. citri] Query ID: lcl|Query_4453 Length: 411

>WP_053884339.1 murein hydrolase activator EnvC [Escherichia coli]
Sequence ID: Query_4455 Length: 427
Range 1: 29 to 427

Score:196 bits(498), Expect:9e-63,
Method:Compositional matrix adjust.,
Identities:143/427(33%), Positives:223/427(52%), Gaps:47/427(11%)

Query  3    LAVGVLACTLLGSMGASAQSQRETERKLQQLRDELKTISAD-------RRELEGKRGTAA  55
            L+ GVL C        SA +           RD+LK+I AD        R+ + +R +
Sbjct  29   LSAGVLLCAF------SAHADE---------RDQLKSIQADIAAKERAVRQKQQQRASLL  73

Query  56   QQLRQADEKVAKTARALSETEAAMRAQEQHLSTLQQERAQLQRGLQNQRAQLAALLRAAD  115
             QL++ +E +++  R L ET+  +    + +  +    A+L++    Q   LAA L AA
Sbjct  74   AQLKKQEEAISEATRKLRETQNTLNQLNKQIDEMNASIAKLEQQKAAQERSLAAQLDAAF  133

Query  116  QVGRNAPLKVLLSQDTVGDATRMLADHRYVQNARAQRIHALTTQLDALATVEQDIATRRQ  175
            + G +  ++++LS +      R+ A   Y+  AR + I  L           +++A +R
Sbjct  134  RQGEHTGIQLILSGEESQRGQRLQAYFGYLNQARQETIAQLKQ-------TREEVAMQRA  186

Query  176  ALDAARAQQKAQAATLQKDRSQQAATVAQLDDRYKQRAEREKAIGQDAKALEQLLAN---  232
             L+  +++Q+     L + R+QQA     L++R K  A  E +I Q  + L +L AN
Sbjct  187  ELEEKQSEQQT---LLYEQRAQQAKLTQALNERKKTLAGLESSIQQGQQQLSELRANESR  243

Query  233  LRAAAAKAEAERRAAARRAAAEAAAQAKRSKTERSDRPGKT--PSKVVANAPAPKVGGLS  290
            LR + A+AEA  +  A R A EA  QA R + + + R G T  P++    +   + GGL
Sbjct  244  LRNSIARAEAAAKVRAEREAREA--QAVRDRQKEATRKGTTYKPTES-EKSLMSRTGGLG  300

Query  291  -------WPVAGNLLARFNATLPDGHTSKGVLIGAPKGTTVTAVADGTVVFSDWMTGYGM  343
                   WPV G  L R+   L      KG++IGA +GT V A+ADG V+ +DW+ GYG+
Sbjct  301  APRGQAFWPVRGPTLHRYGEQLQGELRWKGMVIGASEGTEVKAIADGRVILADWLQGYGL  360

Query  344  ILIVDHGNGYMSLYAHNDTLLRDAGATIKRGDAVAKVGSSGGQGVPALYFELRRNGQPVD  403
            +++V+HG G MSLY +N + L   G+ ++ G  +A VGSSGGQG P+LYFE+RR GQ V+
Sbjct  361  VVVVEHGKGDMSLYGYNQSALVSVGSQVRAGQPIALVGSSGGQGRPSLYFEIRRQGQAVN  420

Query  404  PSSWLQR  410
            P  WL R
Sbjct  421  PQPWLGR  427
```
