# Supplementary material for: EnvC Homolog Encoded by Xanthomonas citri subsp. citri Is Necessary for Cell Division and Virulence
Source: Microorganisms. 2024 Mar 29;12(4):691. doi: 10.3390/microorganisms12040691 (PMC11051873; doi:10.3390/microorganisms12040691)
Supplement: Supplementary file 1 [file microorganisms-12-00691-s001.zip › Supplementary Figure S5.pdf]

Figure S5: Domain multiple sequence alignment of the nine ORFs of *X. citri* sharing the M23 domain. The protein sequences were recovered from the IMG platform and uploaded to the NCBI Batch Web CD-Search tool.

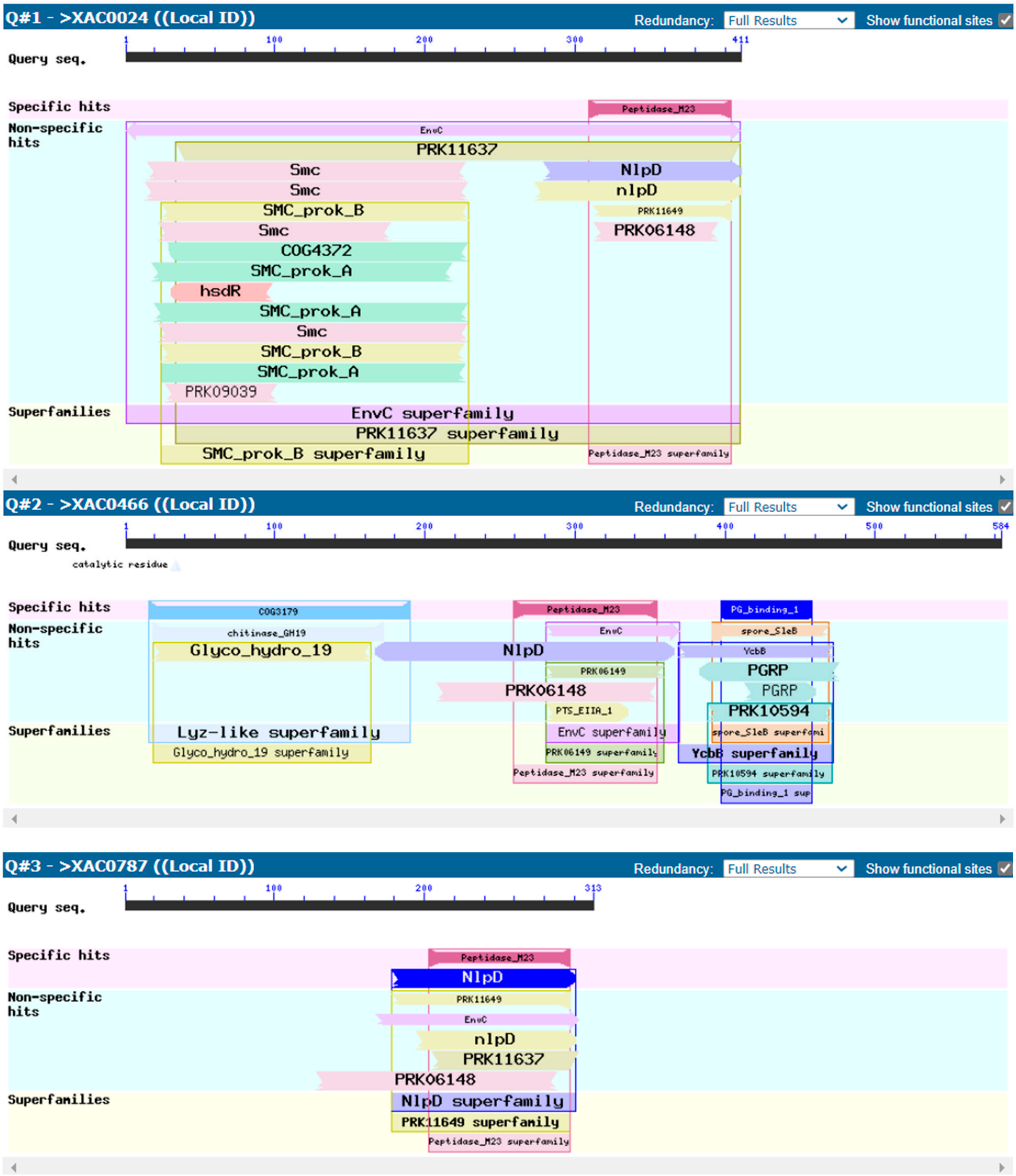

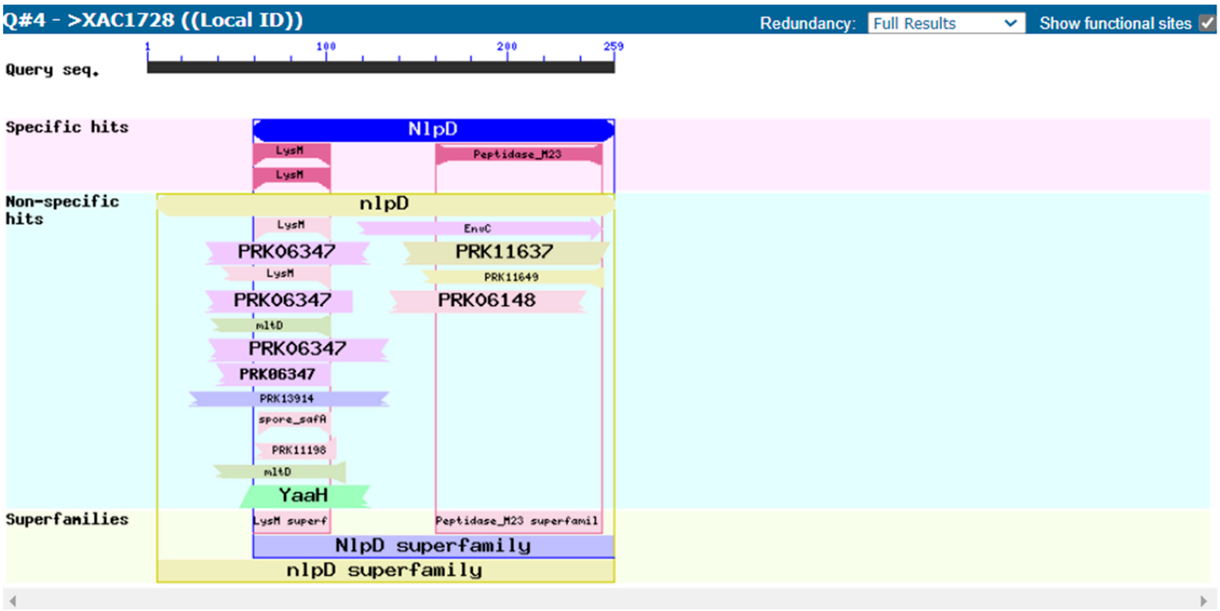
Q#4 - >XAC1728 ((Local ID))
Redundancy: Full Results
Show functional sites
1
100
200
259
Query seq.
Specific hits
NlpD
LysM
LysM
Peptidase_M23
Non-specific hits
nlpD
LysM
EnvC
PRK06347
PRK11637
LysM
PRK11649
PRK06347
PRK06148
mltD
PRK06347
PRK06347
PRK13914
spore_safA
PRK11198
mltD
YaaH
Superfamilies
LysM superf
Peptidase_M23 superfamil
NlpD superfamily
nlpD superfamily

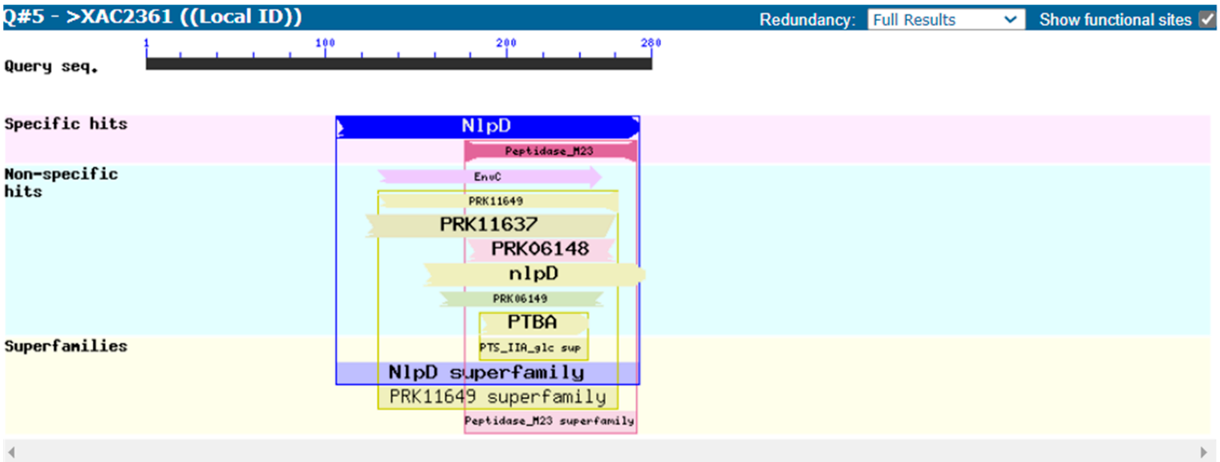
Q#5 - >XAC2361 ((Local ID))
Redundancy: Full Results
Show functional sites
1
100
200
280
Query seq.
Specific hits
NlpD
Peptidase_M23
Non-specific hits
EnvC
PRK11649
PRK11637
PRK06148
nlpD
PRK06149
PTBA
Superfamilies
PTS_IIA_glc sup
NlpD superfamily
PRK11649 superfamily
Peptidase_M23 superfamily

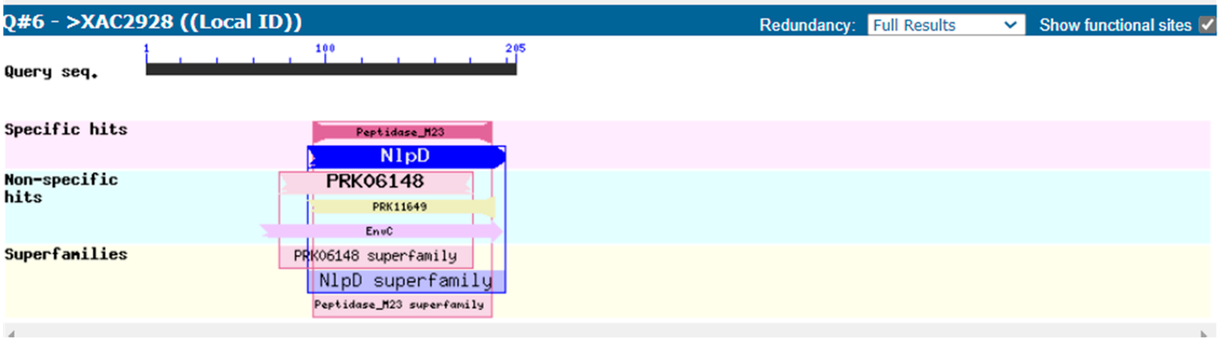
Q#6 - >XAC2928 ((Local ID))
Redundancy: Full Results
Show functional sites
1
100
205
Query seq.
Specific hits
Peptidase_M23
NlpD
Non-specific hits
PRK06148
PRK11649
EnvC
Superfamilies
PRK06148 superfamily
NlpD superfamily
Peptidase_M23 superfamily

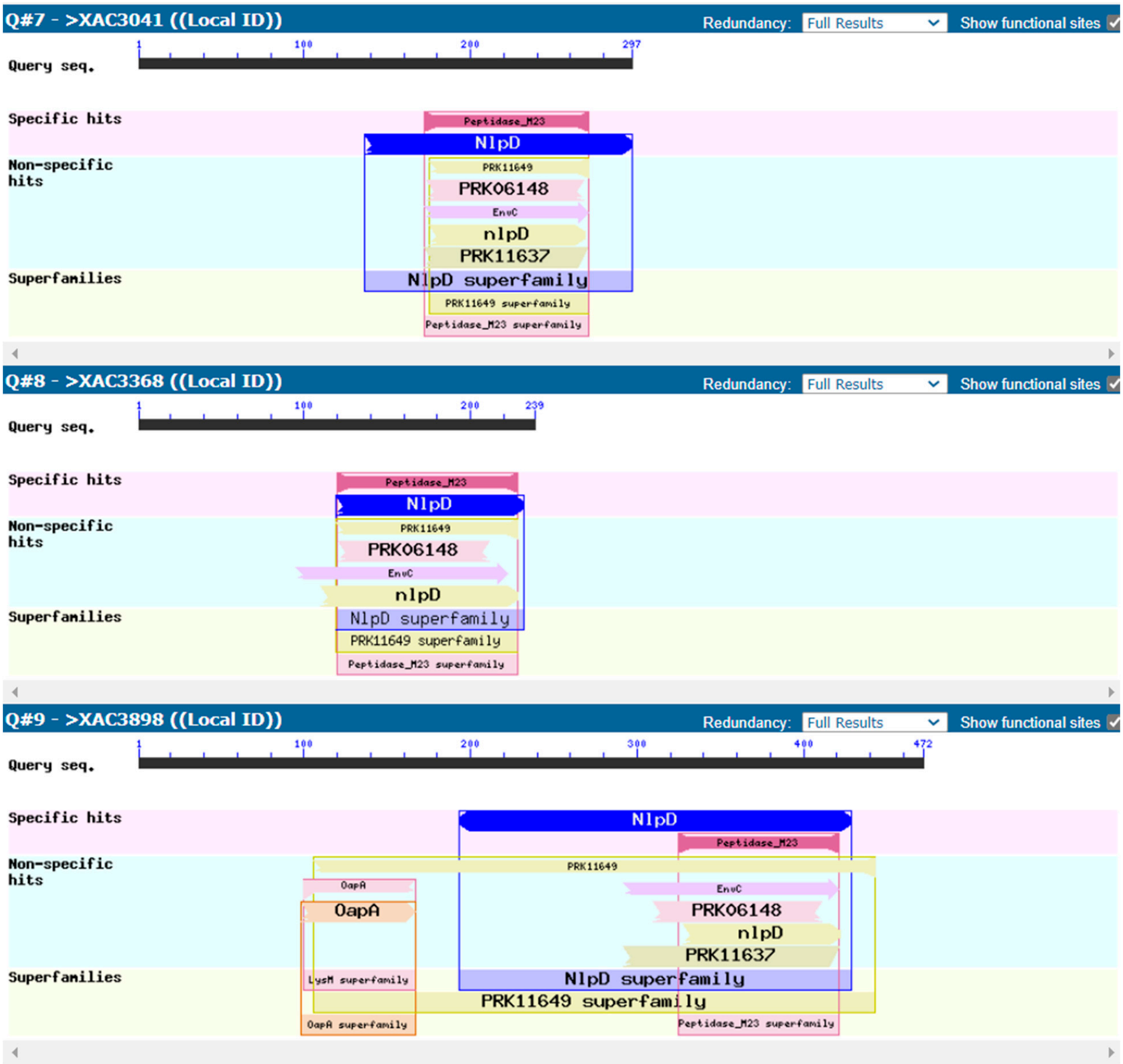
Q#7 - >XAC3041 ((Local ID))
Redundancy: Full Results
Show functional sites
Query seq.
1
100
200
297
Specific hits
Peptidase_M23
NlpD
Non-specific hits
PRK11649
PRK06148
EnvC
nlpD
PRK11637
Superfamilies
NlpD superfamily
PRK11649 superfamily
Peptidase_M23 superfamily
Q#8 - >XAC3368 ((Local ID))
Redundancy: Full Results
Show functional sites
Query seq.
1
100
200
239
Specific hits
Peptidase_M23
NlpD
Non-specific hits
PRK11649
PRK06148
EnvC
nlpD
Superfamilies
NlpD superfamily
PRK11649 superfamily
Peptidase_M23 superfamily
Q#9 - >XAC3898 ((Local ID))
Redundancy: Full Results
Show functional sites
Query seq.
1
100
200
300
400
472
Specific hits
NlpD
Peptidase_M23
Non-specific hits
PRK11649
OapA
OapA
EnvC
PRK06148
nlpD
PRK11637
Superfamilies
LysM superfamily
NlpD superfamily
PRK11649 superfamily
OapA superfamily
Peptidase_M23 superfamily
