# Supplementary material for: EnvC Homolog Encoded by Xanthomonas citri subsp. citri Is Necessary for Cell Division and Virulence
Source: Microorganisms. 2024 Mar 29;12(4):691. doi: 10.3390/microorganisms12040691 (PMC11051873; doi:10.3390/microorganisms12040691)
Supplement: Supplementary file 1 [file microorganisms-12-00691-s001.zip › Supplementary Figure S6.pdf]

Figure S6: Domain multiple sequence alignment of 16 representative species within the *Xanthomonas* species used in the Maximum Likelihood phylogeny. The protein sequences were recovered from the IMG platform and uploaded to the NCBI Batch Web CD-Search tool.

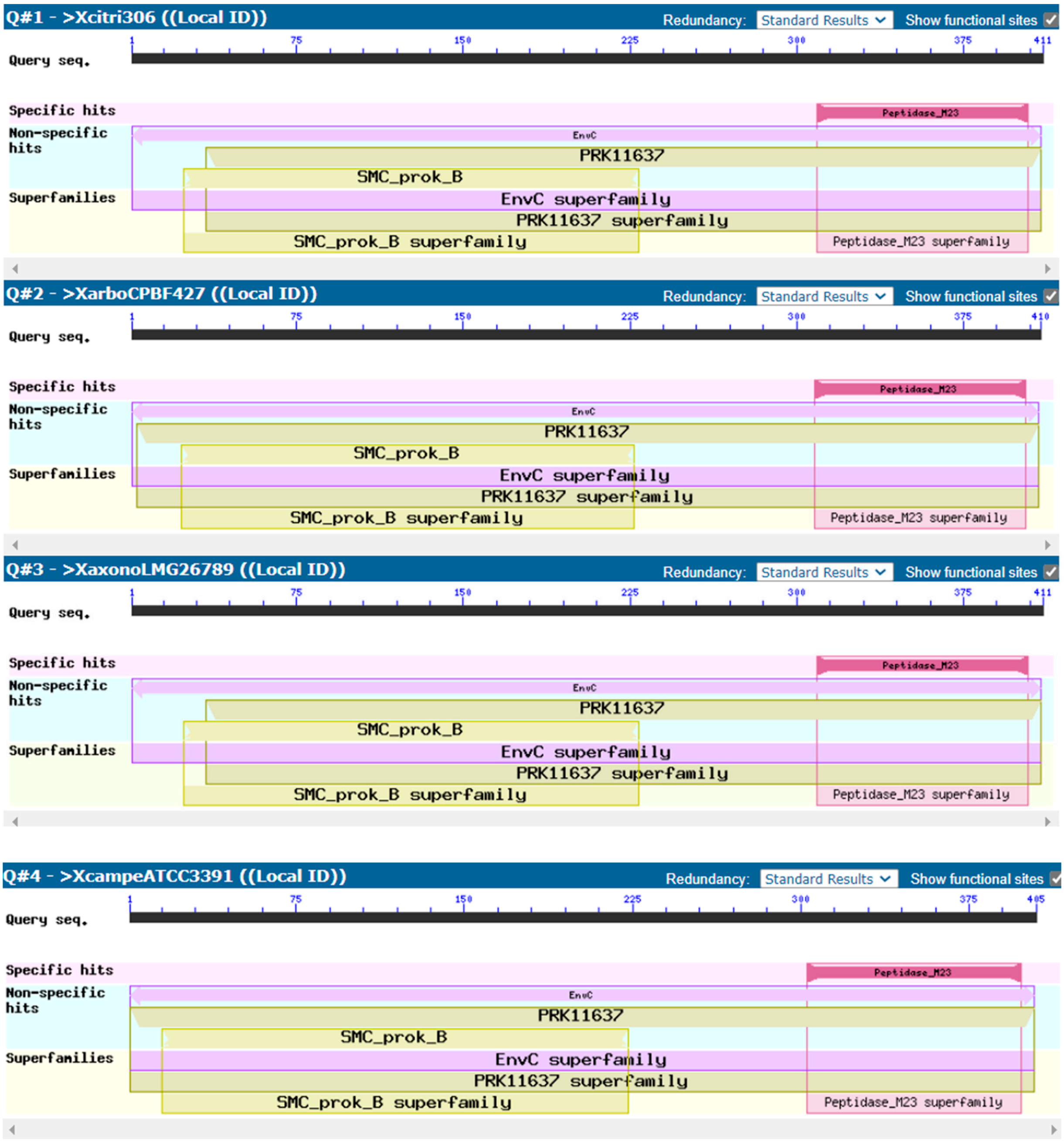

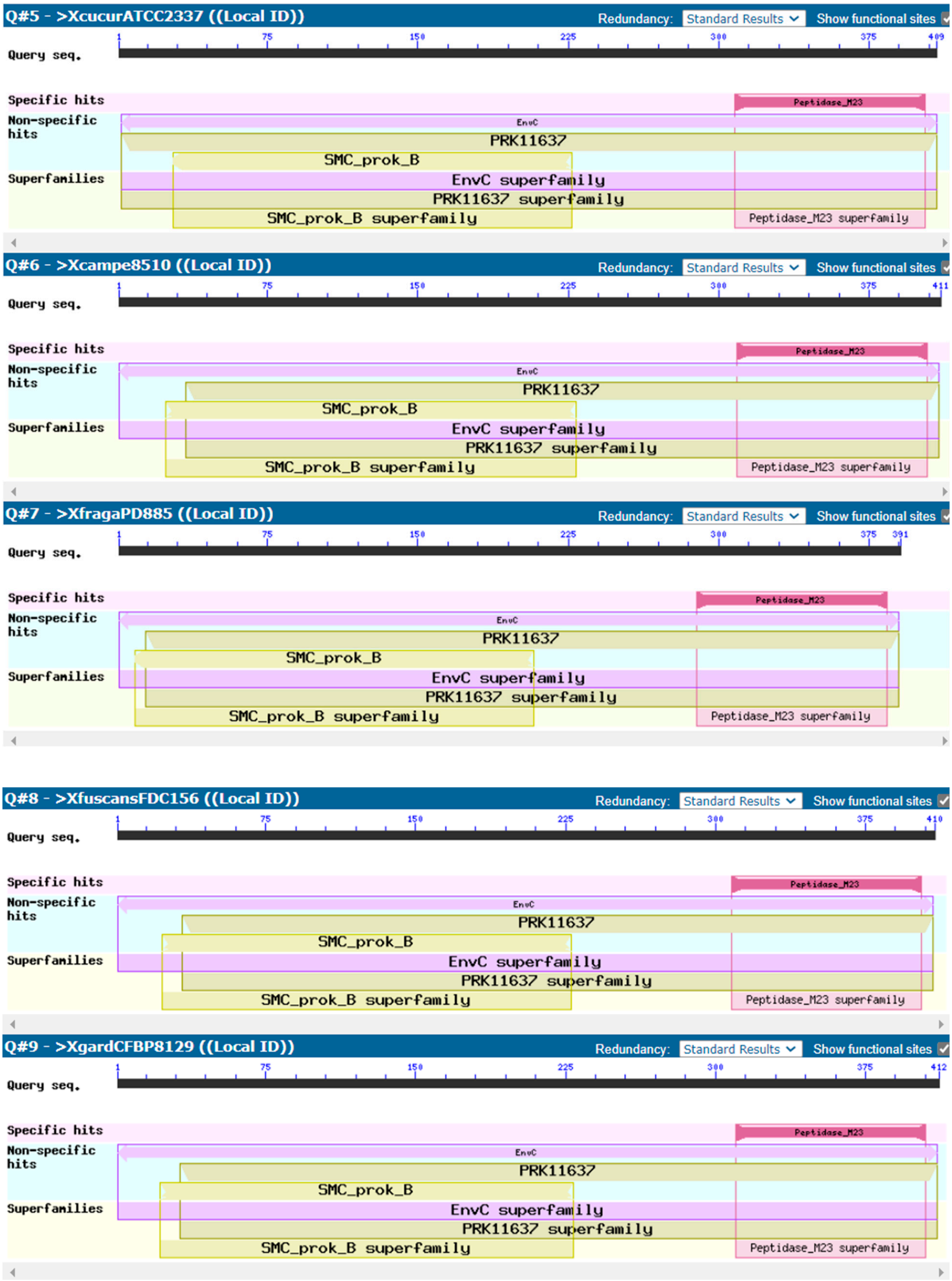
Q#5 - >XcucurATCC2337 ((Local ID))
Redundancy: Standard Results
Show functional sites
Query seq.
1 75 150 225 300 375 409
Specific hits
Non-specific hits
Superfamilies
Peptidase_M23
EnvC
PRK11637
SMC_prok_B
EnvC superfamily
PRK11637 superfamily
SMC_prok_B superfamily
Peptidase_M23 superfamily
Q#6 - >Xcampe8510 ((Local ID))
Redundancy: Standard Results
Show functional sites
Query seq.
1 75 150 225 300 375 411
Specific hits
Non-specific hits
Superfamilies
Peptidase_M23
EnvC
PRK11637
SMC_prok_B
EnvC superfamily
PRK11637 superfamily
SMC_prok_B superfamily
Peptidase_M23 superfamily
Q#7 - >XfragaPD885 ((Local ID))
Redundancy: Standard Results
Show functional sites
Query seq.
1 75 150 225 300 375 391
Specific hits
Non-specific hits
Superfamilies
Peptidase_M23
EnvC
PRK11637
SMC_prok_B
EnvC superfamily
PRK11637 superfamily
SMC_prok_B superfamily
Peptidase_M23 superfamily
Q#8 - >XfuscansFDC156 ((Local ID))
Redundancy: Standard Results
Show functional sites
Query seq.
1 75 150 225 300 375 410
Specific hits
Non-specific hits
Superfamilies
Peptidase_M23
EnvC
PRK11637
SMC_prok_B
EnvC superfamily
PRK11637 superfamily
SMC_prok_B superfamily
Peptidase_M23 superfamily
Q#9 - >XgardCFBP8129 ((Local ID))
Redundancy: Standard Results
Show functional sites
Query seq.
1 75 150 225 300 375 412
Specific hits
Non-specific hits
Superfamilies
Peptidase_M23
EnvC
PRK11637
SMC_prok_B
EnvC superfamily
PRK11637 superfamily
SMC_prok_B superfamily
Peptidase_M23 superfamily

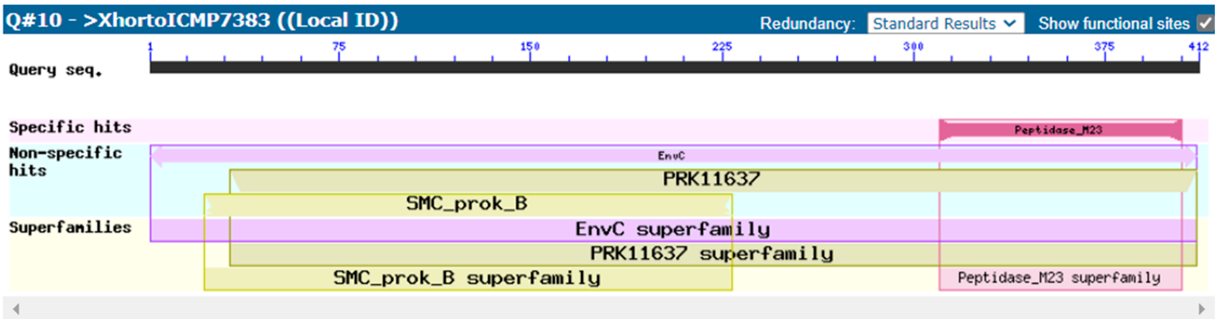
Q#10 - >XhortoICMP7383 ((Local ID))
Redundancy: Standard Results
Show functional sites
1 75 150 225 300 375 412
Query seq.
Specific hits
Non-specific hits
Superfamilies
Peptidase_M23
EnvC
PRK11637
SMC_prok_B
EnvC superfamily
PRK11637 superfamily
SMC_prok_B superfamily
Peptidase_M23 superfamily

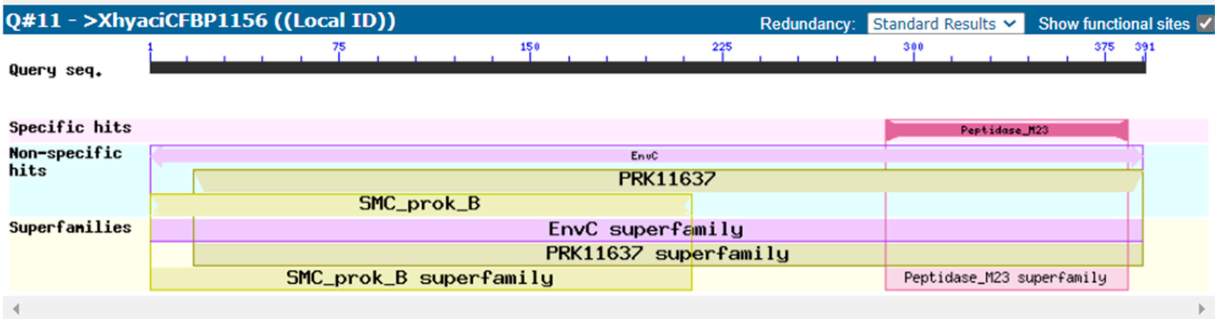
Q#11 - >XhyaciCFBP1156 ((Local ID))
Redundancy: Standard Results
Show functional sites
1 75 150 225 300 375 391
Query seq.
Specific hits
Non-specific hits
Superfamilies
Peptidase_M23
EnvC
PRK11637
SMC_prok_B
EnvC superfamily
PRK11637 superfamily
SMC_prok_B superfamily
Peptidase_M23 superfamily

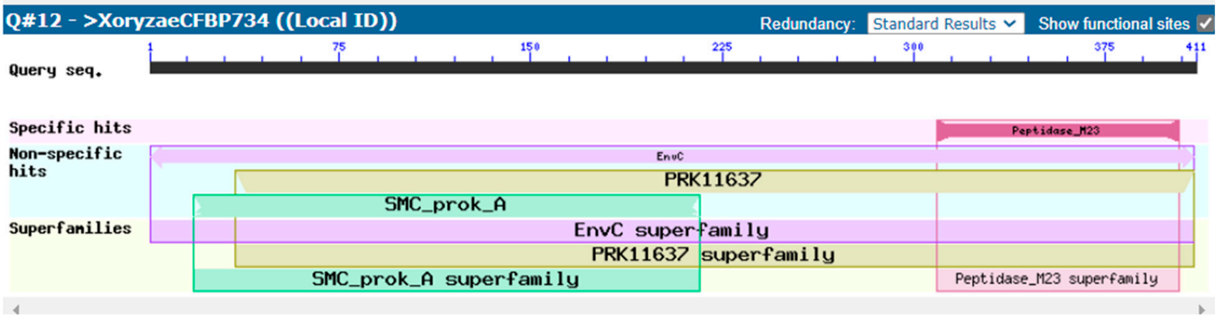
Q#12 - >XoryzaeCFBP734 ((Local ID))
Redundancy: Standard Results
Show functional sites
1 75 150 225 300 375 411
Query seq.
Specific hits
Non-specific hits
Superfamilies
Peptidase_M23
EnvC
PRK11637
SMC_prok_A
EnvC superfamily
PRK11637 superfamily
SMC_prok_A superfamily
Peptidase_M23 superfamily

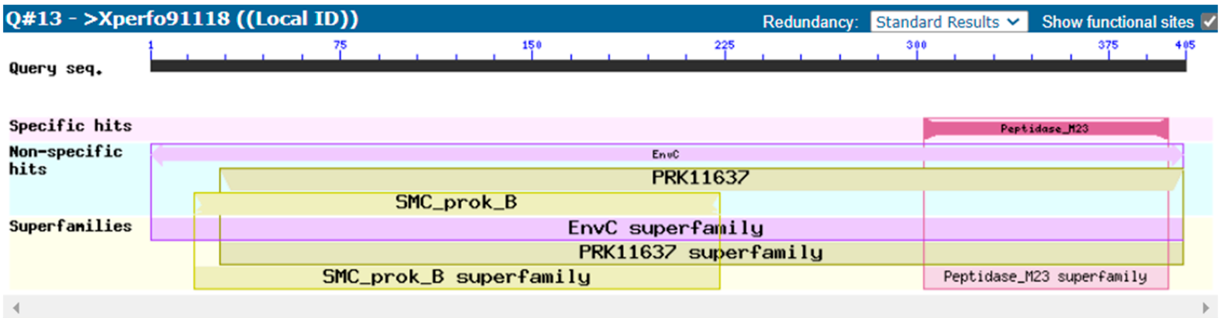
Q#13 - >Xperfo91118 ((Local ID))
Redundancy: Standard Results
Show functional sites
1 75 150 225 300 375 405
Query seq.
Specific hits
Non-specific hits
Superfamilies
Peptidase_M23
EnvC
PRK11637
SMC_prok_B
EnvC superfamily
PRK11637 superfamily
SMC_prok_B superfamily
Peptidase_M23 superfamily

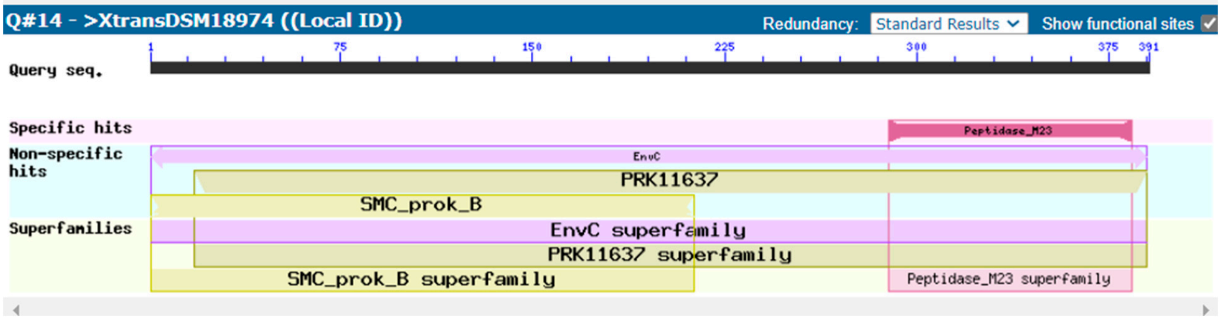
Q#14 - >XtransDSM18974 ((Local ID))
Redundancy: Standard Results
Show functional sites
1 75 150 225 300 375 391
Query seq.
Specific hits
Non-specific hits
Superfamilies
Peptidase_M23
EnvC
PRK11637
SMC_prok_B
EnvC superfamily
PRK11637 superfamily
SMC_prok_B superfamily
Peptidase_M23 superfamily

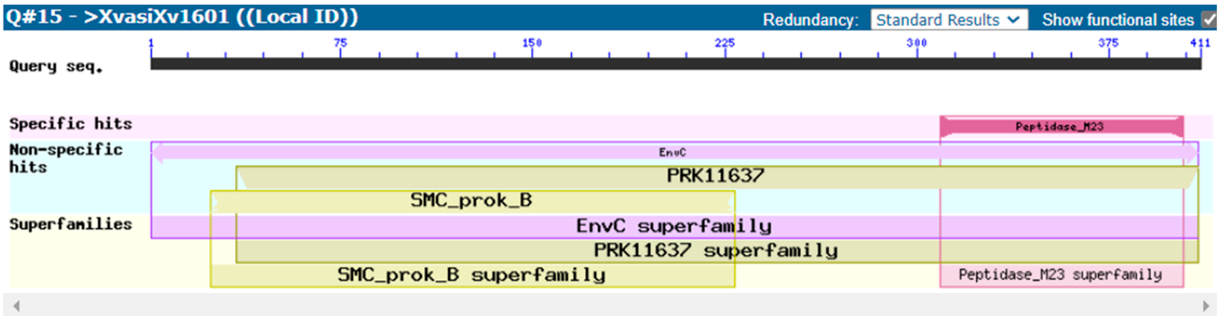
Q#15 - >XvasiXv1601 ((Local ID))
Redundancy: Standard Results
Show functional sites
1
75
150
225
300
375
411
Query seq.
Specific hits
Non-specific hits
Superfamilies
Peptidase_M23
EnvC
PRK11637
SMC_prok_B
EnvC superfamily
PRK11637 superfamily
SMC_prok_B superfamily
Peptidase_M23 superfamily

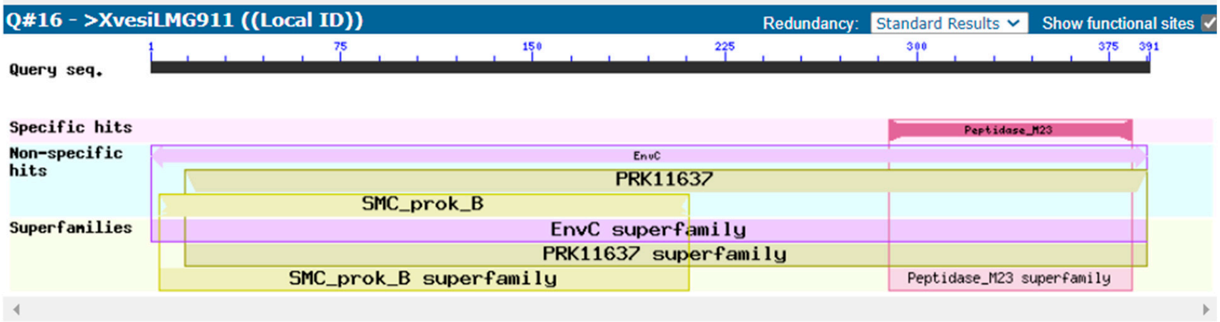
Q#16 - >XvesiLMG911 ((Local ID))
Redundancy: Standard Results
Show functional sites
1
75
150
225
300
375
391
Query seq.
Specific hits
Non-specific hits
Superfamilies
Peptidase_M23
EnvC
PRK11637
SMC_prok_B
EnvC superfamily
PRK11637 superfamily
SMC_prok_B superfamily
Peptidase_M23 superfamily
