# Supplementary material for: EnvC Homolog Encoded by Xanthomonas citri subsp. citri Is Necessary for Cell Division and Virulence
Source: Microorganisms. 2024 Mar 29;12(4):691. doi: 10.3390/microorganisms12040691 (PMC11051873; doi:10.3390/microorganisms12040691)

Figure S7: The predicted signal peptide for XAC0024 from *X. citri*, EnvC from *E. coli* and XCC0022 from *X. campestris*: A) *X. citri XAC0024*; B) *E. coli* EnvC; C) *X. campestris* XCC0022. The analysis was performed using the SignalP 5.0 Server (ARMENTEROS et al., 2019).

A

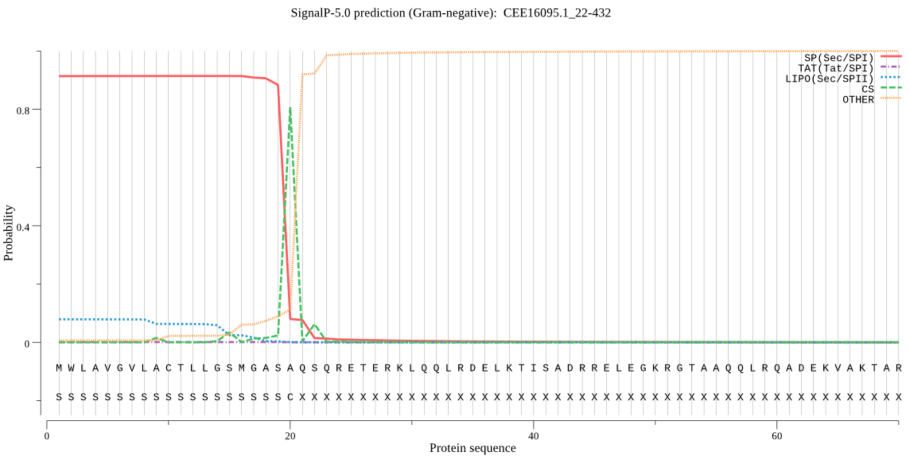

B

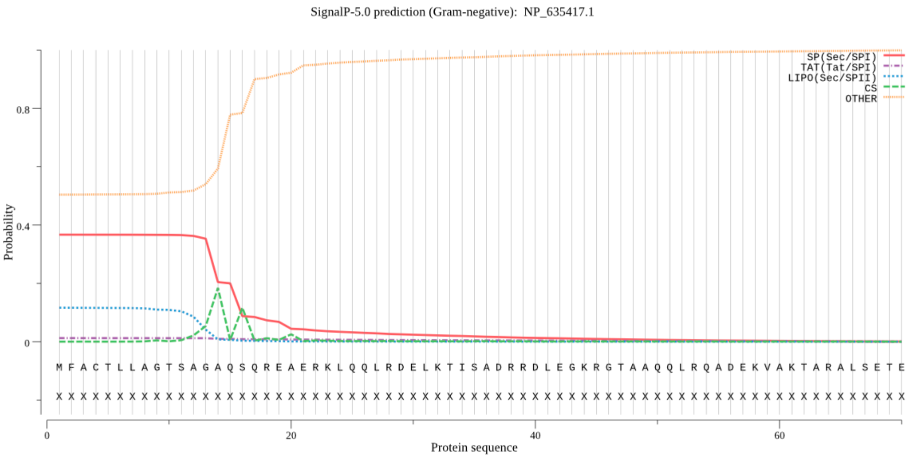

C

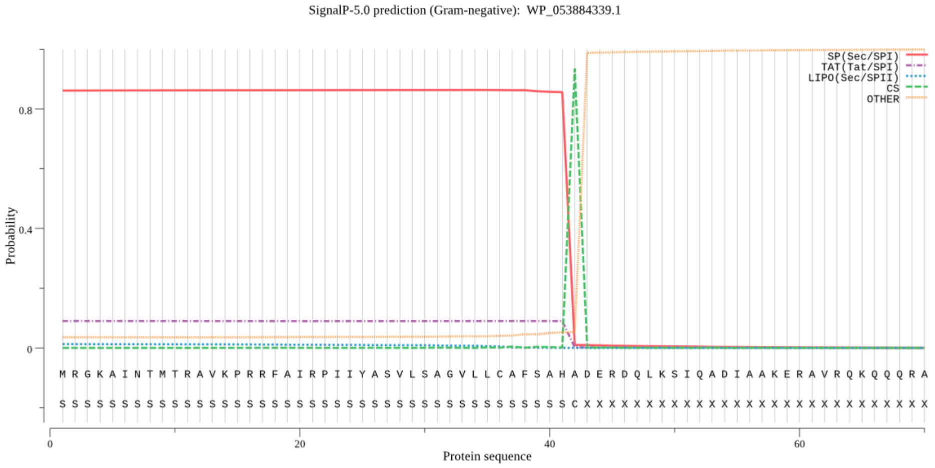

Supplement: Supplementary file 1 [file microorganisms-12-00691-s001.zip › Supplementary Figure S7.pdf]
