# Supplementary material for: EnvC Homolog Encoded by Xanthomonas citri subsp. citri Is Necessary for Cell Division and Virulence
Source: Microorganisms. 2024 Mar 29;12(4):691. doi: 10.3390/microorganisms12040691 (PMC11051873; doi:10.3390/microorganisms12040691)
Supplement: Supplementary file 1 [file microorganisms-12-00691-s001.zip › Supplementary Table S1_S2_S3_S4.pdf]

Table S1: Proteins of *Xanthomonas citri* subsp. *citri* 306 strain sharing the Peptidase M23 Domain according to an *in-silico* search using the IMG 'find function' tool (img.jgi.doe.gov).

Locus Tag	IMG Gene ID	Original Gene Product Name	Signal Peptide	Amino acid Residue	Length bp	Pfam ID
XAC0024	637293275	hypothetical protein	Yes	411	1236	pfam01551 Peptidase_M23
XAC0466	637293717	lytic enzyme	No	584	1755	pfam01551 Peptidase_M23
XAC0787	637294037	peptidase	No	313	942	pfam01551 Peptidase_M23
XAC1728	637294977	lipoprotein	Yes	259	780	pfam01551 Peptidase_M23
XAC2361	637295610	peptidase	Yes	280	843	pfam01551 Peptidase_M23
XAC2928	637296177	peptidase	No	205	618	pfam01551 Peptidase_M23
XAC3041	637296290	hypothetical protein	Yes	297	894	pfam01551 Peptidase_M23
XAC3368	637296617	hypothetical protein	No	239	720	pfam01551 Peptidase_M23
XAC3898	637297147	hypothetical protein	No	472	1419	pfam01551 Peptidase_M23

Table S2: List of strains and plasmids used in this work.

Strains	Characteristics	References
X. citri 306	*Xanthomonas citri* subsp. *citri* strain 306 (wild-type strain)	IBSBF 1594; [14]
Δ*envC*	*X. citri envC* deletion mutant (deletion of genomic bases 26156 to 26748)	This work
E. coli DH10B	Cloning strain	Invitrogen, Waltham, MA, USA
E. coli SM10λpir	Cloning strain	Laboratory Stock
E. coli HST08	Cloning strain	Takara Bio USA, Inc. Mountain View, CA, USA
**Plasmids**	**Characteristics**	**References**
pGEM® -T easy	Cloning vector; Ap^R	Promega
pOK1	*sacB-sacR*; Sp^R	[15]
pMAJIIc	Derivative of pGCD21; mCherry expression vector; Ap^R; Neo^R/Km^R; *ara*C-p*ara;* integrative vector in *X. citri*;	[16] (GenBank MT119765)

Ap^R: ampicillin resistance; Km^R: kanamycin resistance; Neo^R: neomycin resistance; Sp^R: spectinomycin resistance

Table S3: Primers used in this study.

**Primer Name**	**Sequence (5' → 3')**
A(F)	CAGAGCCAGCGCGAGACCGAG
B(R)	<u>ACGGTGGTGCAGCGGTGC</u>ATTGCGGCCCAC
C(F)	<u>GCACCGCTGCACCACCGT</u>GACTGCAGTGGC
D(R)	TCAGCGGCGTTGCAGCCAGCT
0024_500_IF_F	AGATCCATGGCACTCGAGCGCCACTGATCTGGC
0024_500_IF_R	GCTCACCATCTCGAGGTCGTCGATCGGCGCGATCA
0024_IF_F	AGATCCATGGCACTCGAGATGTGGCTGGCGGTG
0024_IF_R	GCTCACCATCTCGAGGCGGCGTTGCAGCCAGCTCGA
M13/pUC F-20	GTAAAACGACGGCCAGT
M13/pUC R-48	AGCGGATAACAATTTCACACAGGA
pGCD1-F	CACACTTTGCTATGCCATAGC
XAC0024_mcherry-F	GTCTCGAGATGTGGCTGGCGGTGGGCGTG

F: forward primer, R: reverse primer.

Underlined bases: sequences that enable the double-joint of fragments A-B and C-B by PCR

Table S4: *Xanthomonas* species used for the phylogenetic reconstruction based on the XAC0024 nucleotide sequence of *X. citri*.

**Species name**	**amino acid residues**	**GenBank accession no.**	**Genome position**	**% of identity**
Xanthomonas arboricola pv. *juglandis* CPBF 427	422	LR824643.1	24462..25695	89%
Xanthomonas arboricola pv. *pruni* strain 15-088	405	CP044334.1	2764090..2765323	89%
Xanthomonas axonopodis pv. *commiphoreae* strain LMG26789	411	CP031059.1	25792..27027	96%
Xanthomonas axonopodis pv. *vasculorum* strain NCPPB 796	411	CP053649.1	25983..27218	96%
Xanthomonas campestris pv. *badrii* strain NEB122	425	CP051651.1	4480484..4481713	88%
Xanthomonas campestris pv. *campestris* str. ATCC 33913	405	AE008922.1	25781..27013	85%
Xanthomonas campestris pv. *musacearum* NCPPB 4379	411	CP034655.1	24291..25526	93%
Xanthomonas campestris pv. *raphani* strain MAFF106181	429	CP058243.1	25980..27212	85%
Xanthomonas campestris pv. *vesicatoria* str. 85-10	411	CP017190.1	3549491..3550726	96%
Xanthomonas citri pv. *glycines* strain EB08	411	CP026334.1	25875..27110	99%
Xanthomonas citri pv. *malvacearum* strain MS14003	411	CP023159.1	25878..27113	99%
Xanthomonas citri pv. *phaseoli* var. *fuscans* strain CFBP6991	411	CP021015.1	2719200..2720435	98%

Xanthomonas citri pv. *punicae* strain LMG 859	411	CP030178.1	928824..930059	99%
Xanthomonas citri pv. *vignicola* strain CFBP7113	411	CP022270.1	27974..29206	98%
Xanthomonas cucurbitae strain ATCC 23378	420	CP033326.1	23211..24440	87%
Xanthomonas euvesicatoria strain LMG930	411	CP018467.1	4550833..4552068	96%
Xanthomonas fragariae strain PD885	439	LT853882.1	28773..30007	87%
Xanthomonas fuscans subsp. *aurantifolii* strain FDC 1561	411	CP011250.1	26157..27389	98%
Xanthomonas gardneri strain CFBP 8129	413	LR828253.1	23961..25200	88%
Xanthomonas hortorum pv. *gardneri* strain ICMP 7383	440	CP018731.1	90415..91654	88%
Xanthomonas hortorum pv. *gardneri* strain JS749-3	440	CP018728.1	544060..545299	88%
Xanthomonas hyacinthi strain CFBP 1156	407	CP043476.1	1562009..1563187	75%
Xanthomonas oryzae pv. *oryzicola* strain CFBP7342	432	CP007221.1	34791..36026	93%
Xanthomonas oryzae strain NCPPB 4346	411	CP036253.1	25678..26913	93%
Xanthomonas perforans 91-118	405	CP019725.1	2777398..2778633	96%
Xanthomonas perforans strain LH3	411	CP018475.1	3366136..3367371	96%
Xanthomonas translucens pv. *cerealis* strain 01	409	CP038228.1	4306080..4307258	75%
Xanthomonas translucens pv. *translucens* DSM 18974	437	LT604072.1	471751..472929	74%

Xanthomonas vasicola pv. *vasculorum* strain Xv1601	411	CP025272.1	24271..25506	93%
Xanthomonas vesicatoria ATCC 35937 strain LMG911	405	CP018725.1	2926304..2927535	90%
